# Understanding Early Neuropsychiatric Symptoms in Alzheimer's Disease Using REDGESAM: A Study Focused on Depression and Anxiety

**DOI:** 10.1002/alz70857_101375

**Published:** 2025-12-24

**Authors:** Carlos Orellana, Ixim Anibal Morales, Jorge Ochoa

**Affiliations:** ^1^ GBHI, San Francisco, CA, USA; ^2^ Universidad San Sebastian, Santiago, Santiago, Chile

## Abstract

**Background:**

Neuropsychiatric symptoms (NPS), such as depression and anxiety, are common in the early stages of Alzheimer's disease (AD). These symptoms often appear before cognitive decline becomes evident, posing significant challenges for patients and caregivers. Despite their prevalence, the underlying mechanisms of NPS remain poorly understood, particularly in underserved populations.

**Method:**

A retrospective cohort study will utilize REDGESAM's electronic clinical records, focusing on patients aged 50 and older diagnosed with mild cognitive impairment (MCI) or subjective cognitive decline (SCD). Data sources include standardized neuropsychiatric scales and brain imaging reports. Advanced statistical analyses, including regression modeling, will assess associations between NPS, imaging findings, and cognitive decline.

**Result:**

This study anticipates identifying key correlations between NPS and brain imaging markers such as hippocampal volume and white matter integrity. These findings will provide a deeper understanding of how early psychiatric symptoms align with biological changes in the brain.

**Conclusion:**

By leveraging REDGESAM's robust data, this research will inform targeted interventions for depression and anxiety in AD, improve patient outcomes, and contribute to dementia care strategies worldwide

